# Uncovering the diagnostic potential of seminal fluid beyond fertility: cfDNA methylation analysis for the detection of clinically significant prostate cancer

**DOI:** 10.1371/journal.pone.0356387

**Published:** 2026-09-09

**Authors:** Jennifer Geis, James F. Smith, Tara Maddala, Greg Thoreson, Kiarash Michel, Jed Kaminetsky, Laurence Belkoff, Stephanie Huang, Bailey Griscom, James C. Hart, Shellie Bench, Hamisha Ardalani, Laura Rivas-Yepes, Daniel Civello, Kim M. Clark-Langone

**Affiliations:** 1 Fellow Health Inc., San Leandro, California, United States of America; 2 Dept. of Urology, University of California, San Francisco, California, United States of America; 3 Department of Clinical Research, Urology Clinics of North Texas, Dallas, Texas, United States of America; 4 Comprehensive Urology, Los Angeles, California, United States of America; 5 Integrated Medical Professionals, New York, New York, United States of America; 6 MidLantic Urology, Bala Cynwyd, Pennsylvania, United States of America; Nelson Mandela African Institute of Science and Technology, TANZANIA, UNITED REPUBLIC OF

## Abstract

Research on the potential use of seminal fluid as a liquid biopsy for prostate cancer detection has been limited due to challenges associated with acquisition of this bodily fluid in clinical studies. Here we sought to expand on our previous analysis, which demonstrated high levels of prostate-derived cell free DNA (cfDNA) in seminal fluid in presumed healthy individuals, to a much larger cohort that included participants with prostate cancer. A total of 279 men scheduled for prostate biopsy were enrolled over 4 months across 12 sites. Prior to their biopsy, participants mailed a seminal fluid sample collected at home to the laboratory, from which cfDNA was extracted and underwent methylation analysis. Consistent with our earlier study in healthy individuals, we observed an abundance of high molecular weight (HMW) cfDNA in all samples. Tissue-of-origin deconvolution revealed that granulocytes and sperm were the principal contributors to seminal fluid cfDNA, while prostate-derived cfDNA was present at abundances readily detectable with current technologies. The nucleosomal fraction was very pronounced in some but not all samples and was determined to be correlated with the relative sperm signal. The sperm signal was also observed to be associated with an increase in small insert sizes (< 125 bp) in the sequenced libraries. Unsupervised clustering revealed two distinct populations driven by the abundance of sperm and granulocytes. Since summarizing at the genomic region level confounded tissues of different origins, fragment-level DNA methylation features were used to characterize and quantify the prostate cancer related signal, and features associated with clinically significant prostate cancer were identified. This study expands on our previous work to further characterize seminal fluid and highlights its potential as a promising liquid biopsy medium for the detection and monitoring of clinically significant prostate cancer.

## Introduction

Prostate cancer is the most common solid organ malignancy in men, accounting for an estimated 35,200 deaths in 2024 [1]. Screening for prostate cancer using prostate-specific antigen (PSA) began in the 1980s. Despite a consistent annual decline in per capita mortality, the use of PSA testing remains controversial due to its questionable efficacy in identifying lethal prostate cancer. PSA testing has demonstrated a reduction in prostate cancer mortality of 21% in the ERSPC trial [2] while the PLCO trial showed no significant benefit [3], likely due to widespread opportunistic screening in the control group. However, PSA testing is limited by poor specificity ranging from 13% to 29% [4] which contributes to high false-positive rates, with only one in four men with elevated PSA levels ultimately diagnosed with prostate cancer. Moreover, PSA testing lacks the ability to reliably differentiate between indolent tumors (Grade Group <2) and clinically significant disease (Grade Group 2+), and its performance is significantly influenced by inflammation and infection – factors that can elevate PSA levels independent of cancer. Autopsy studies have revealed that a substantial proportion of men harbor histologic evidence of prostate cancer without ever experiencing symptoms during their lifetime, highlighting the potential harms of screening and treatment in cases of indolent disease [5]. Biomarkers such as PCA3, the 4-kallikrein panel, TMPRSS2:ERG fusion gene, microRNAs, androgen receptor variants, and PTEN inactivation have shown promise in liquid biopsies but remain constrained by sensitivity limitations of blood and urine [5,6].

Since prostatic fluid contributes 37–44% of seminal fluid volume [7] it has been proposed that biomarkers, including prostate-derived tumor cells, in seminal fluid could lead to the emergence of novel and non-invasive diagnostic approaches for prostate cancer [8,9] potentially offering high specificity without compromising on sensitivity. However, due to the sensitive nature of seminal fluid collection and subsequent instability of cell free nucleic acids, sample acquisition has historically been difficult, and research using this bodily fluid for liquid biopsy has been slow to evolve. We recently demonstrated that cell free DNA (cfDNA) in seminal fluid can be preserved, opening the door for home collection [10] that is both non-invasive and convenient.

Previous studies suggested that higher amounts of high-molecular-weight (HMW) cfDNA in seminal fluid were associated with prostate cancer [11,12]. Additionally, our recent study (in presumed healthy individuals) demonstrated that the prostate signal is higher in HMW cfDNA compared to the nucleosomal cfDNA fraction, and that prostate-derived cfDNA can make up more than 20% of the total HMW cfDNA in seminal fluid from men without vasectomies. Despite our recent advancements in understanding the tissue of origin of cfDNA in seminal plasma in presumed healthy controls [10], there are no large studies describing the molecular composition of cfDNA in seminal plasma from men suspected of having prostate cancer.

Epigenetic alterations, particularly aberrant DNA methylation, have emerged as critical biomarkers for cancer detection due to their early and pervasive occurrence during tumorigenesis and their stability across diverse biological samples [13–16]. Cancer cells are characterized by global hypomethylation and focal hypermethylation of CpG islands, especially in the promoters of tumor suppressor genes, which can be detected at early neoplastic stages and serve as highly specific and sensitive indicators of malignancy [13,17]. Recent advances in molecular technologies have enabled the analysis of methylation signatures in blood, urine, and other body fluids, facilitating early diagnosis and ongoing monitoring of many cancer types [14,18,19]. However, sensitivity remains in the single digits for the detection of early disease [19,20] and there has been limited success in the detection of prostate cancer using circulating tumor DNA in blood. This is largely because in early-stage prostate cancer, tumor DNA is not abundantly shed into the bloodstream. Given our previous results where we demonstrated that prostate derived cfDNA was abundant in seminal fluid in assumed healthy controls [10], we sought to determine whether prostate cancer could be detected in seminal fluid using DNA methylation markers. Moreover, we aimed to establish if clinically significant prostate cancer (sigPCa) could be distinguished from indolent or no prostate cancer which could facilitate a diagnostic test that has both high sensitivity and specificity.

The objectives of this study were to characterize methylation profiles of cfDNA in men undergoing prostate biopsy for suspicion of prostate cancer. We demonstrate that granulocytes and sperm are the predominant sources of cfDNA in seminal fluid and that prostate cfDNA is at abundances measurable by technologies available today. By using methylation pattern analysis, we identified methylation biomarkers that were associated with clinically significant prostate cancer. These findings pave the way for seminal fluid-based at home diagnostics as a transformative tool in prostate cancer management.

## Materials and methods

### Clinical study information

Specimens were collected from participants enrolled in the **PR**ostate cancer b**I**omarker detection in **S**eminal plas**M**a (PRISM) clinical study. The study received ethical approval by an Institutional Review Board (IRB00000533); IRB tracking number 20232059. Written informed consent was obtained either electronically or on paper from study participants prior to engaging in study activities. Participants were enrolled from 12 sites from 11/21/23 to 3/15/24 and were required to be over 40 years of age, scheduled to undergo a prostate biopsy within 2 months and able to ejaculate a semen specimen. Exclusion criteria included a prior diagnosis of prostate cancer, any treatment for other cancer within 1 year (except treatment for non-melanoma skin cancer which was considered acceptable), any invasive urologic procedures within the last 3 months, any 5 alpha reductase inhibitors within 3 months, any alpha-blocker use within 2 weeks, and prior participation in the study (i.e., participants could not be enrolled more than once). Clinical history, including PSA, DRE status, MRI status, current cancer diagnosis and pathology was collected using an electronic data capture system (EDC).

### Specimen collection

Upon enrollment, a collection kit was sent to the subject’s home. Participants were asked to refrain from ejaculation for 2–5 days before specimen collection and to provide their semen specimen prior to their scheduled biopsy. Any specimen produced after biopsy was rejected. Participants were instructed to produce their specimen by masturbation into a collection cup, mix with the provided preservative and ship to Fellow Health’s molecular R&D laboratory the same day. Participants were requested to write the time and day of ejaculation, the time the specimen was mixed with the preservative and the number of days of abstinence.

### Seminal fluid processing

Upon receipt, specimens were rejected if they were not received within 5 days of ejaculation or if the semen specimen did not contain preservative. The volume of the specimen was logged in addition to abstinence time and whether the specimen was visually viscous. Any specimen that was less than 500 µl volume and had no visible cell pellet was rejected. (Note that 500 µl of preservative was provided so at least 500 µl was expected to be returned). The seminal fluid was first spun at 400 x g for 15 mins at room temperature to pellet sperm and somatic cells. The supernatant was transferred to a new tube and spun at 16,000 x g to pellet cellular debris and protamine associated cfDNA from mature sperm. The supernatant was then transferred to a new tube and stored at -80C.

### cfDNA extraction and size selection

Total cell free nucleic acid was extracted as previously described [10] and eluted from the column in 100 µl of elution buffer. Up to 60 µl underwent size selection as previously described [10] to obtain small size selected (SSD) cfDNA and HMW cfDNA. The HMW cfDNA was subjected to 250 seconds of sonication using a Covaris ultrasonicator (peak incident power, 50 W; duty factor, 20%; cycles per burst, 200; temperature, 20 °C.), followed by a SPRI clean up using 1.5x ratio of SPRI reagent to sample volume.

### Library preparation and sequencing

Paired methylation libraries were prepared for each sample using up to 20 ng SSD and up to 40 ng fragmented HMW using New England Biolab’s NEBNext® Enyzmatic Methyl-Seq kit, according to the manufacturer’s instructions. Target enrichment was then performed using Twist’s Human Methylome Panel, according to the manufacturer’s instructions. All libraries were sequenced on a NovaSeqX (either on a 10B or 25B flowcell), 2 x 150 bp reads, at a target of 260M - 300M reads per sample.

### Sequencing data processing and analysis

Demultiplexing was performed and reads were trimmed to remove adapters and low quality sequences using fastp v0.23.4 with the extra options: ‘--trim_poly_g -f 1’ (20). Reads were aligned to GRCh38 using bwa-meth v0.2.7 [21] and bwa-mem2 v2.2.1 [22]. Following alignment, reads were sorted and indexed using samtools v1.3 [23], and read duplicates were marked using Picard tools v3.1.0 (http://broadinstitute.github.io/picard). CpGs were then quantified for the number of methylated and unmethylated bases using MethylDackel v0.6.1 (https://github.com/dpryan79/MethylDackel) and beta-values (fraction of methylated bases) were calculated for each methylation location.

The CpG beta-values were then summarized across ~512,000 regions of the methylome and used to assess similarity between samples using unsupervised hierarchical clustering. Additionally, tissue deconvolution was performed on the PRISM samples to quantify the relative fractions of sperm, prostate epithelial, bladder epithelial, kidney epithelial, kidney endothelial, B-cells, T-cells, NK-cells, granulocytes, and monocytes for each sample. The same methodology and reference matrix were used from our previous publication (2), and the results were compared with the tissue deconvolution results from the healthy individuals.

Samples were assessed for quality based on typical quality metrics such as q30 base rate, estimated diversity, on target rate, insert size, coverage uniformity, and GC bias. Pre-defined thresholds based on previously processed samples were used, and samples failing any of these metrics were excluded from further analysis.

### Clinically significant prostate cancer biomarker identification and modeling

All data analyses followed a predefined Statistical Analysis Plan (SAP) covering data management, processing, and analysis protocols. Clinical data was cleaned and locked separately from assay data quality control and lock. Data from each remained blinded prior to merging and analysis.

Our first attempt to identify biomarkers related to clinically significant prostate cancer followed traditional methods for DMR (differentially methylated region) testing using the summarized beta-values across regions with a false discovery rate correction on the p-values [24,25]. It was determined after a methylome-wide analysis that any DMRs found were actually confounded with background tissue noise and not true biomarkers of clinically significant prostate cancer.

In order to derive biomarkers that were not artifacts of background tissue, the methylome panel was subsetted for known prostate cancer related genes and regions. Read fragments were then sorted and grouped into features based on methylation status similarity and abundance from a training set of N = 150 HMW samples. Because of the large number of features, an empirical approach was taken to reduce the discovery of “false positive” biomarker features. The same training set was randomly split into 5-repeats 10-folds so that each analysis set contained 90% of the training data with a total of 50 different analysis subsets. Each analysis subset was bootstrapped 1000x with replacement, and for each bootstrap, Welch’s t-test p-value was calculated between clinically significant (sigPCa) and non-clinically significant prostate cancer (non-sigPCa) samples. The p-values were then compiled into a distribution for each analysis subset resulting in 50 distributions of p-values for each feature. Features that consistently appeared in all 50 p-value distributions with p-values < 0.05 were used to build a classifier to detect clinically significant prostate cancer. This aggressive approach to preliminary feature selection was taken to identify non-artifactual features that were also highly robust.

Using only the training data, a logistic regression elastic net model was tuned and fitted. A second model was also built and tested in a similar manner while including age and PSA as covariates. Prediction probabilities for both models were then calculated on a completely independent test set of N = 18 samples. Note that the test set was a true hold-out set and was not used in any step of the training processes. The overall performance of the models was then assessed on the test set using ROCs (Receiving Operator Curves) and their AUCs (Area Under the Curves).

All analyses, feature selection, and modeling following bioinformatic read processing was performed in R v4.4.0. Plots were generated using ggplot v3.5.2 [26]. Modeling was performed using caret v7.0-1 [27].

## Results

### Clinical cohort

Specimens were delivered to the laboratory typically the next day (85% were delivered the next day, 93% were delivered within 2 days and anything beyond 6 days was rejected). Of 276 enrolled participants, 60 (22%) did not provide a specimen. This compliance rate of 78% is very favorable compared to published compliance rates for diagnostic procedures [28,29], suggesting that seminal fluid is a convenient biofluid to collect even in this older population. After excluding participants that were also discontinued for various reasons, an additional 13 samples were rejected either based on QC criteria or insufficient cfDNA from extraction. This resulted in a total of 171 samples that were used to create both an SSD and HMW library for methylation sequencing. See S1 Fig for additional details. Table 1 shows participant demographics and other metrics of the 171 samples that went through methylation sequencing.

**Table 1:** Summary of demographics and clinical information for N = 171 samples that were sequenced.

		Total	Negative	Positive = 102				
				Grade Grp 1	Grade Grp 2	Grade Grp 3	Grade Grp 4	Grade Grp 5
		N = 171	N = 69	N = 41	N = 30	N = 17	N = 11	N = 3
**Age**	Mean (SD)	63.7 (7.1)	61.9 (8)	63.9 (5.9)	65.4 (6.7)	66.2 (6.9)	64.9 (4.4)	66.0 (10.0)
	Median (IQR)	64.0 (9.5)	62.0 (11.0)	64.0 (5.0)	67.0 (6.0)	67.0 (7.0)	65.0 (5.5)	66.0 (10.0)
**Race/ Ethnicity**	White, n (%)	135 (78.9%)	50 (72.5%)	37 (90.2%)	24 (80.0%)	14 (82.4%)	8 (72.7%)	2 (66.7%)
	Black, n (%)	18 (10.5%)	7 (10.1%)	2 (4.9%)	4 (13.3%)	2 (11.8%)	3 (27.3%)	0 (0%)
	Other, n (%)	9 (5.3%)	7 (10.1%)	1 (2.4%)	0 (0%)	0 (0%)	0 (0%)	1 (33.3%)
	NA, n (%)	9 (5.3%)	5 (7.3%)	1 (2.4%)	2 (6.7%)	1 (5.9%)	0 (0%)	0 (0%)
**Vasectomy**	Yes, n (%)	22 (12.9%)	5 (7.3%)	9 (22.0%)	3 (10.0%)	5 (29.4%)	0 (0%)	0 (0%)
	No, n (%)	147 (86.0%)	63 (91.3%)	32 (78.0%)	27 (90.0%)	12 (70.6%)	10 (90.9%)	3 (100%)
	NA, n (%)	2 (1.2%)	1 (1.5%)	0 (0%)	0 (0%)	0 (0%)	1 (9.1%)	0 (0%)
**Family History of PCa**	Yes, n (%)	46 (26.9%)	20 (29.0%)	14 (34.1%)	6 (20.0%)	3 (17.6%)	3 (27.3%)	0 (0%)
	No, n (%)	115 (67.3%)	44 (63.8%)	23 (56.1%)	23 (76.7%)	14 (82.4%)	8 (72.7%)	3 (100%)
	NA, n (%)	10 (5.9%)	5 (7.3%)	4 (9.8%)	1 (3.3%)	0 (0%)	0 (0%)	0 (0%)
**BPH**	Yes, n (%)	77 (45.0%)	32 (46.4%)	22 (53.7%)	11 (36.7%)	8 (47.1%)	4 (36.4%)	0 (0%)
	No, n (%)	84 (49.1%)	32 (46.4%)	19 (46.3%)	16 (53.3%)	9 (52.9%)	5 (45.5%)	3 (100%)
	NA, n (%)	10 (5.9%)	5 (7.3%)	0 (0%)	3 (10.0%)	0 (0%)	2 (18.2%)	0 (0%)
**Prostatitis**	Yes, n (%)	4 (2.3%)	3 (4.4%)	0 (0%)	0 (0%)	1 (5.9%)	0 (0%)	0 (0%)
	No, n (%)	163 (95.3%)	64 (92.8%)	41 (100%)	29 (96.7%)	16 (94.1%)	10 (90.9%)	3 (100%)
	NA, n (%)	4 (2.3%)	2 (2.9%)	0 (0%)	1 (3.3%)	0 (0%)	1 (9.1%)	0 (0%)
**MRI**	Yes, n (%)	114 (66.7%)	47 (68.1%)	33 (80.5%)	20 (66.7%)	6 (35.3%)	6 (54.5%)	2 (66.7%)
	No, n (%)	54 (31.6%)	20 (29.0%)	8 (19.5%)	9 (30.0%)	11 (64.7%)	5 (45.5%)	1 (33.3%)
	NA, n (%)	3 (1.8%)	2 (2.9%)	0 (0%)	1 (3.3%)	0 (0%)	0 (0%)	0 (0%)
**PSA (ng/mL)**	Mean (SD)	7.3 (6.3)	5.7 (2.8)	6.4 (2.7)	7.9 (7.2)	9.4 (6.4)	14.5 (16.9)	11.2 (4.5)
	Median (IQR)	6.0 (3.9)	5.0 (3.4)	5.8 (4.2)	6.2 (3.3)	7.0 (4.6)	7.1 (7)	10.1 (4.4)

The clinical cohort consisted of a wide variety of participants, despite the large majority being white (78.9%). Data was extremely complete with minimal missing information. Aside from the notable representation of participants with a vasectomy, nearly half of all participants had Benign Prostatic Hyperplasia (BPH); however, the number of patients with prostatitis was minimal. Also of note was the significant number of participants that underwent an MRI. This was likely site driven and due to standard practices of the location. A large range of PSA values were also represented, including N = 29 or 17.0% of participants with PSA levels less than 4 ng/mL (See S2 Fig).

### cfDNA yield

**Fig 1:**
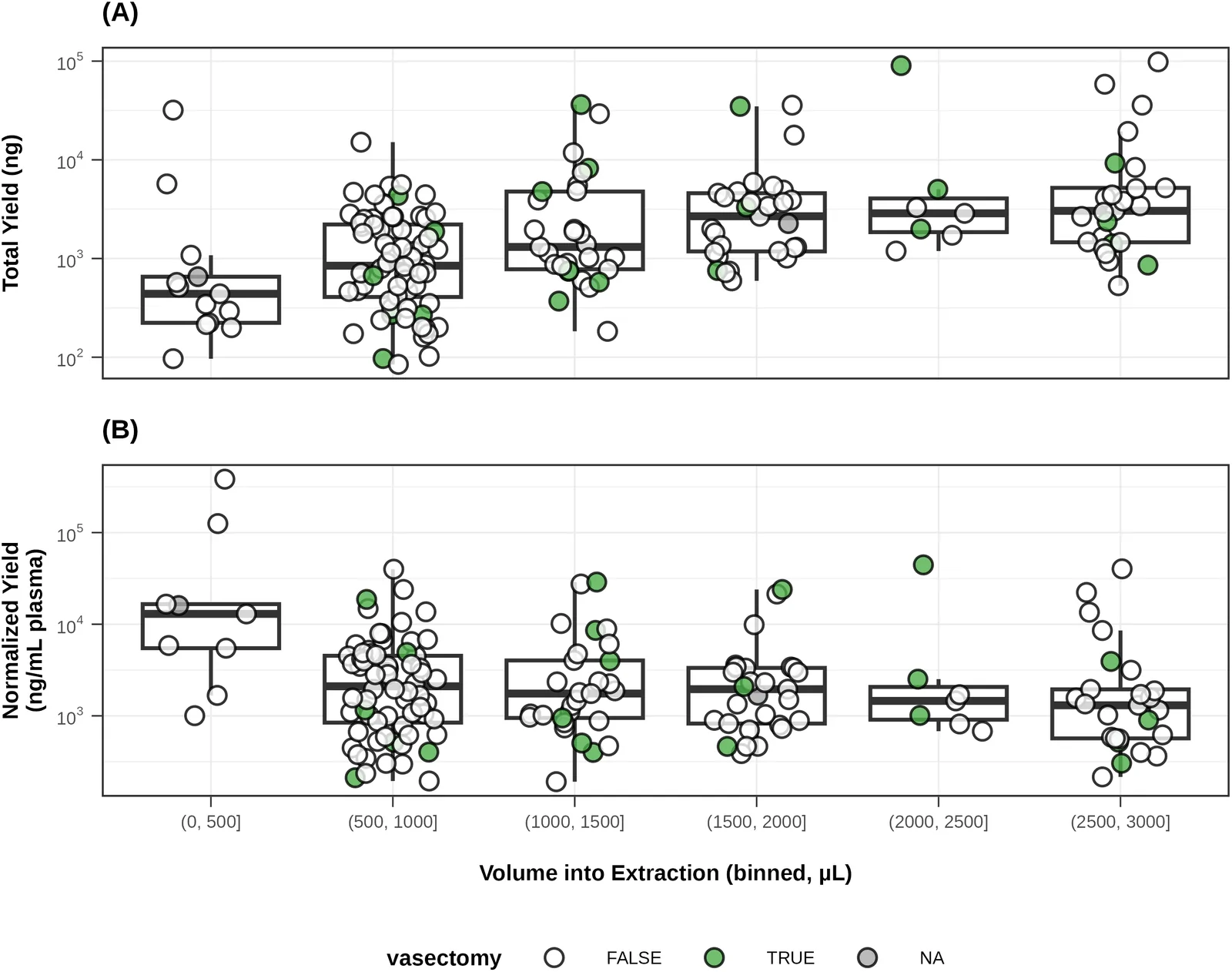
cfDNA yield. A: Total yield (ng) from extraction as a function of the volume (ul) of seminal plasma into extraction. B: Normalized yield (ng per mL of plasma) as a function of the volume (ul) of seminal plasma into extraction.

Fig 1A illustrates total cfDNA yield and Fig 1B illustrates normalized yield (ng per mL of plasma). The mean normalized yield was 7861 ng per mL of plasma, which is over 7 times higher than what was seen in our previous study with (generally younger) healthy individuals [10]. The mean total yield of cfDNA (from 75% of the 100 µl eluate) was 4829 ng. It is noteworthy that vasectomy participants do not stand out as having lower yields of cfDNA (the mean normalized yield was 11,829 ng/mL and mean total yield was 9468 ng). A maximum of 3 mL seminal plasma was used in total nucleic acid extraction. For comparison, in stage I-III cancer patients, cfDNA yield from blood is < 20 ng/mL plasma [30]. In urine, cfDNA yields can vary from 1–200 ng/mL [31].

### DNA Tapestation profiles

**Fig 2:**
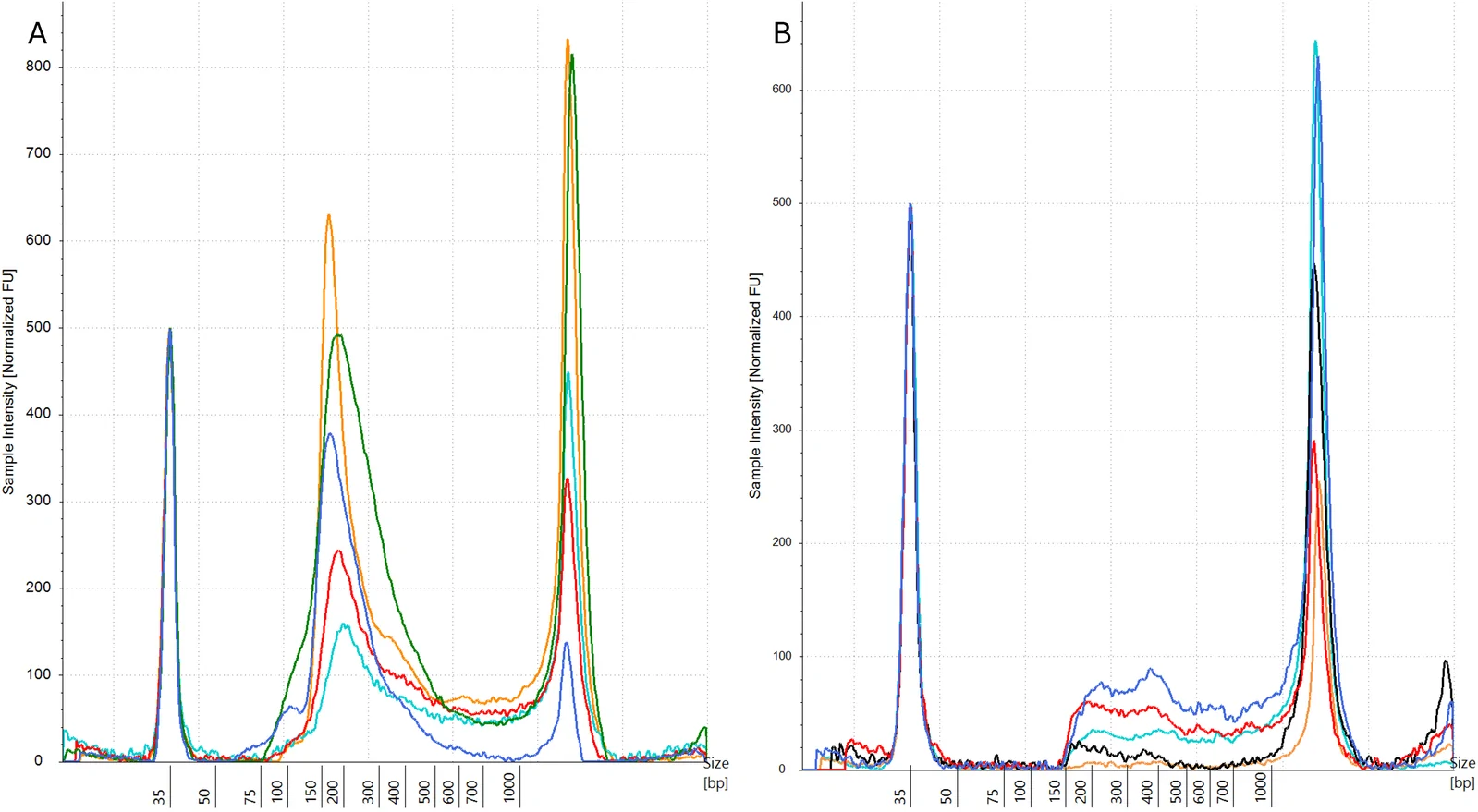
cfDNA Tapestation traces show two different populations, one with a distinct nucleosomal peak and one without. A: Five different participants that all have a clear nucleosomal peak. B: Five different participants, none of which have a distinct nucleosomal peak.

Fig 2 shows the two types of profiles that were commonly observed in this study. In many participants, there was a clear nucleosomal peak in addition to the HMW peak (Fig 2A), while in other participants there was no distinct nucleosomal peak (Fig 2B).

### Tissue of origin deconvolution

**Fig 3:**
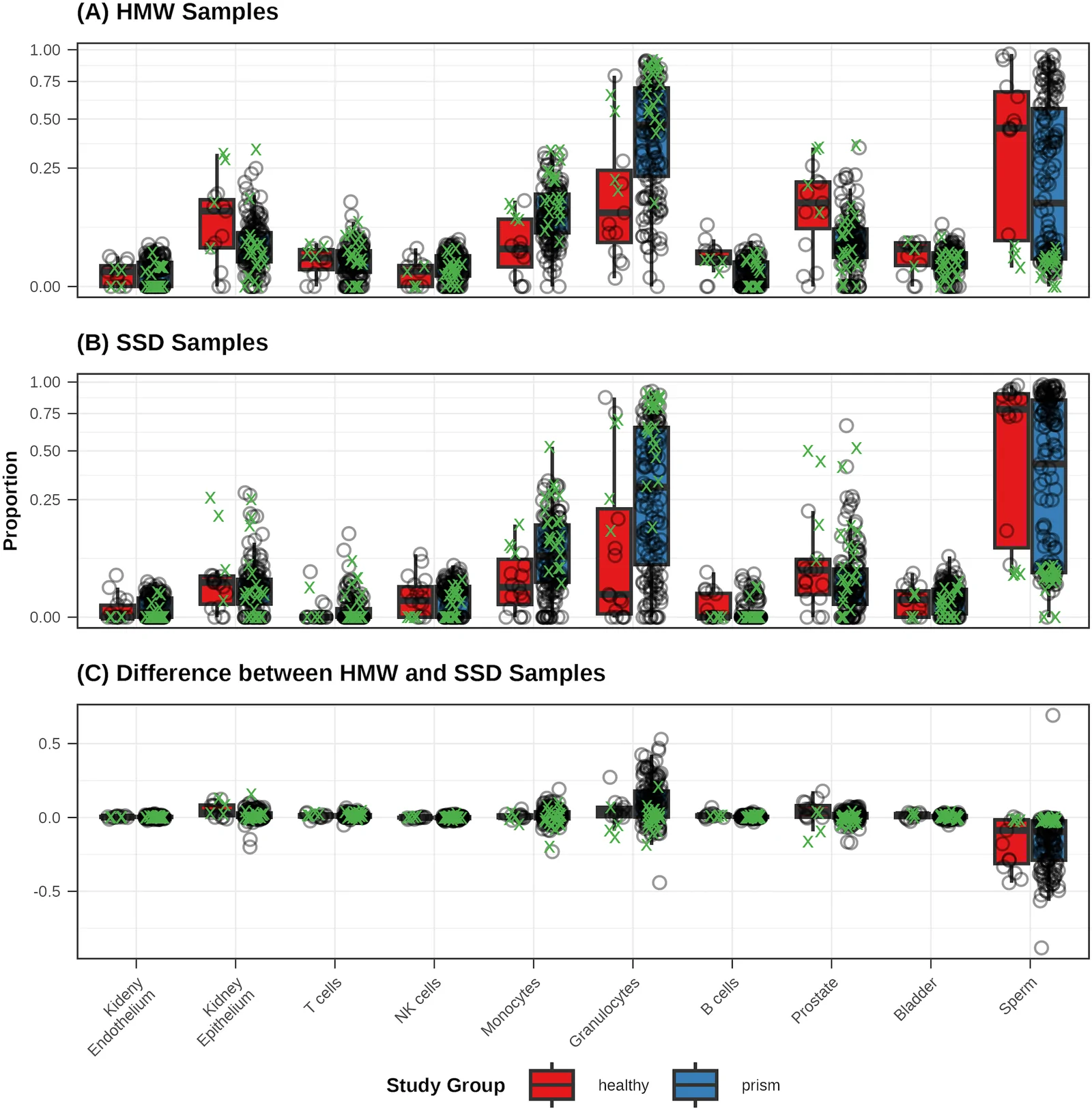
Tissue deconvolution distribution results illustrate that PRISM samples have a much higher abundance of granulocyte signal compared to healthy individuals and that HMW has a lower sperm signal compared to SSD in non-vasectomy men. A: HMW. B: SSD. C: The difference of HMW – SSD. Samples from men with a vasectomy indicated by green “X”. The y-axis is on the square-root scale.

The results of the tissue of origin deconvolution from healthy individuals from our previous publication [10] was compared against the results from these PRISM samples. The distributions between the two studies are shown for HMW (Fig 3A) and SSD (Fig 3B) with samples from men with a vasectomy indicated by green “X’s”. The paired difference between HMW and SSD is also shown (Fig 3C).

**Fig 4:**
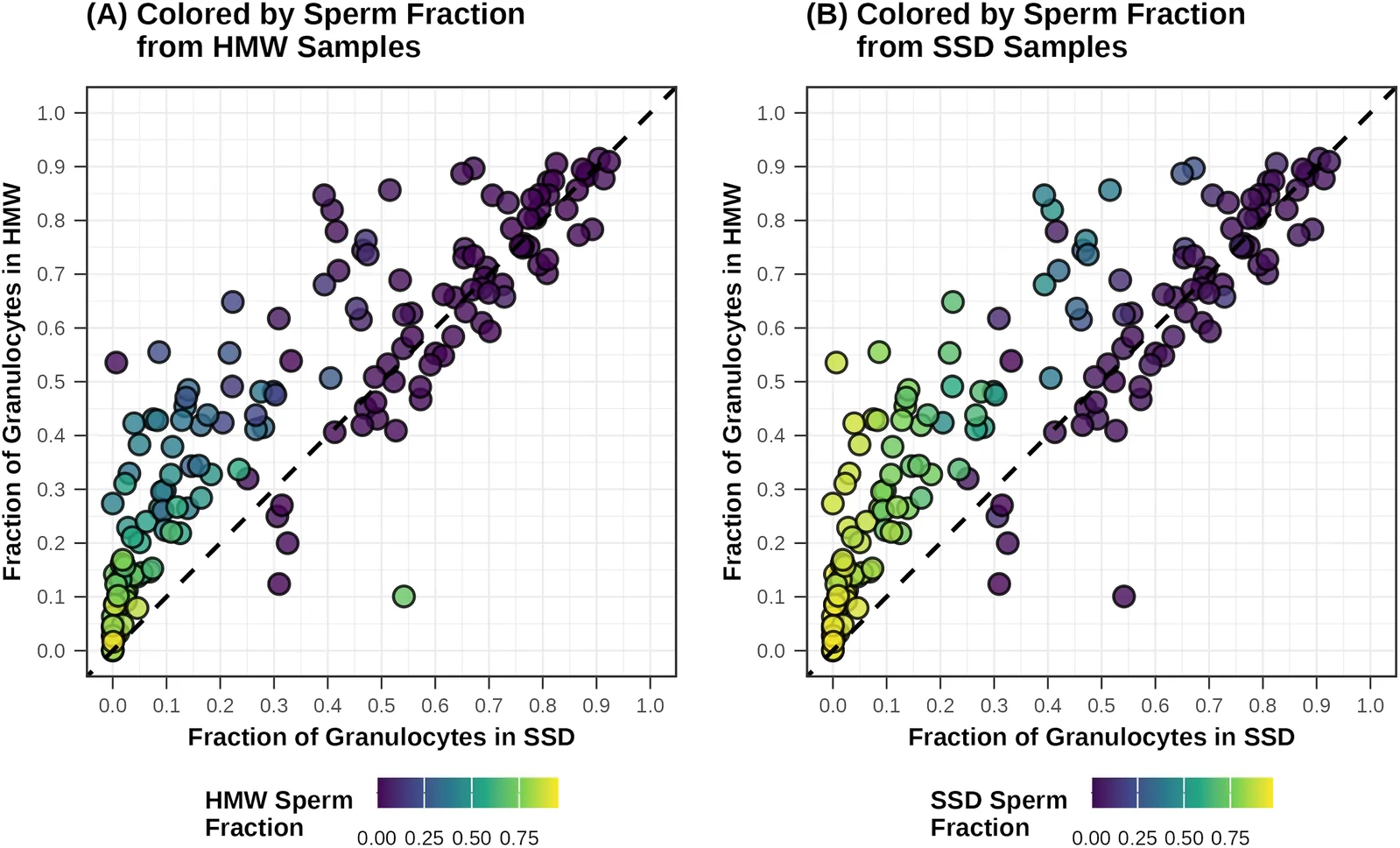
Fraction of granulocyte signal in HMW vs. SSD as a function of sperm signal. A: Colored by the HMW sperm fraction. B: Colored by the SSD sperm fraction.

There are clear distribution similarities for men with vasectomies between both studies for HMW and SSD. Notable is the large number of samples with a high granulocyte signal that were part of the PRISM study. We also observed that the granulocyte signal was higher in HMW than SSD, especially in participants not lacking sperm signal (Fig 4).

Since the tissue deconvolution algorithm ensures the proportions sum to 1, it is not a measure of absolute signal. To determine whether the low sperm signal seen in many non-vasectomy participants was a technical artifact of this, the normalized yield (ng per mL plasma) as a function of sperm and granulocyte fraction was assessed. As shown in S2 Fig, non-vasectomy participants with very high granulocyte signal did not always have higher normalized yields than non-vasectomy participants with very low granulocyte signal. In other words, if the high granulocyte participants had “normal levels” of sperm cfDNA, but also an abundance of granulocyte cfDNA, then the normalized cfDNA yield would be generally higher.

### cfDNA length distribution in Tapestation and insert sizes

**Fig 5:**
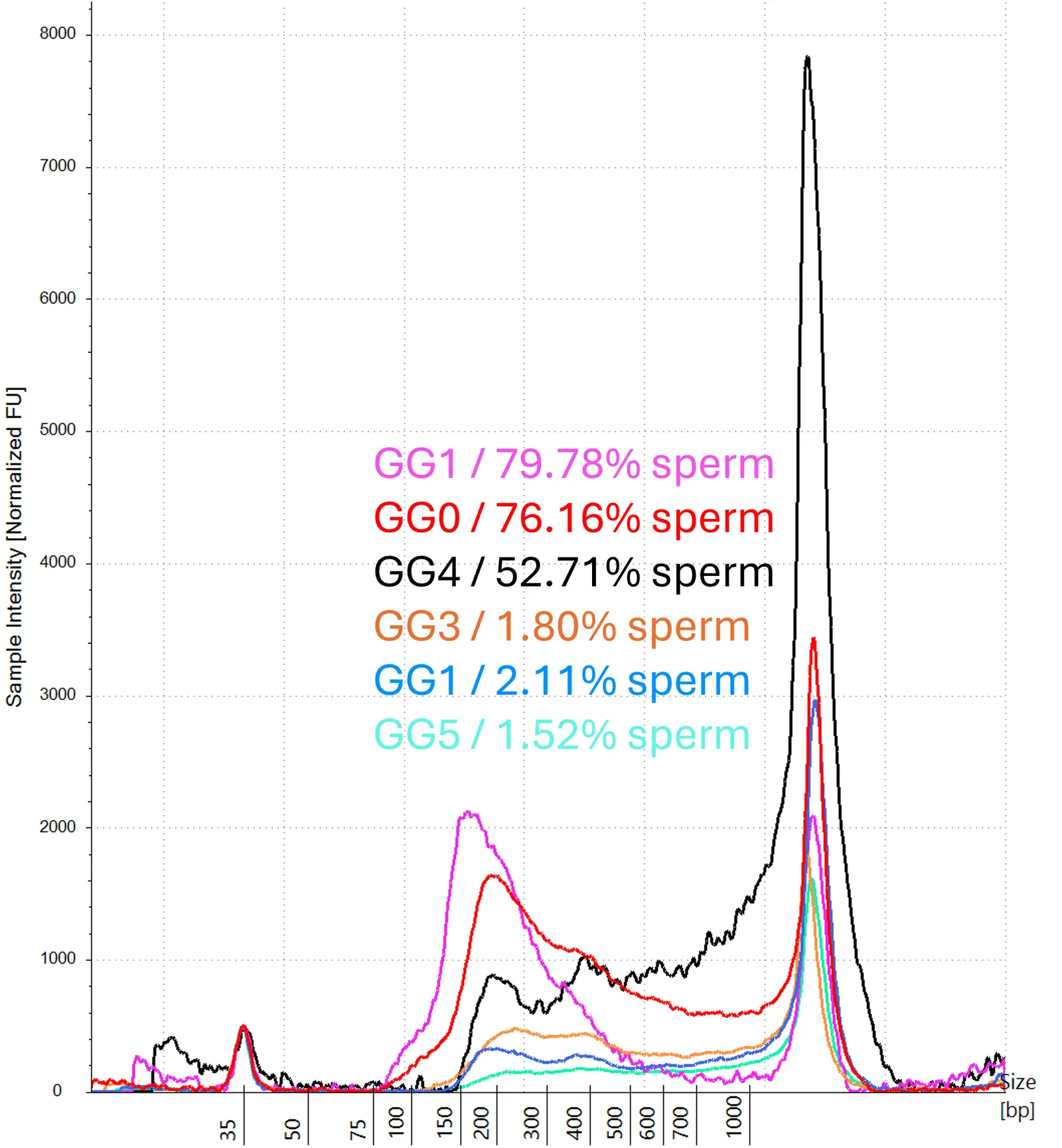
Tapestation traces illustrate that nucleosomal peak size is associated with the amount of sperm signal. The presence/absence of the nucleosomal peak was much more associated with the fraction of (SSD) sperm signal than PCa GG. In this example, the sample with the highest fraction of sperm had the highest nucleosomal peak.

After obtaining tissue of origin information, we examined the Tapestation traces and noticed that the presence/absence of the nucleosomal peak was associated with the fraction of (SSD) sperm signal (see Fig 5). This is again consistent with our previous observations that men with vasectomies appear to lack a distinct nucleosomal peak. However, it should be noted that this phenomenon was also observed in men without vasectomies in both our previous study [10] and the PRISM study. The presence/absence of the nucleosomal peak did not appear to correlate with prostate cancer Grade Group (GG).

**Fig 6:**
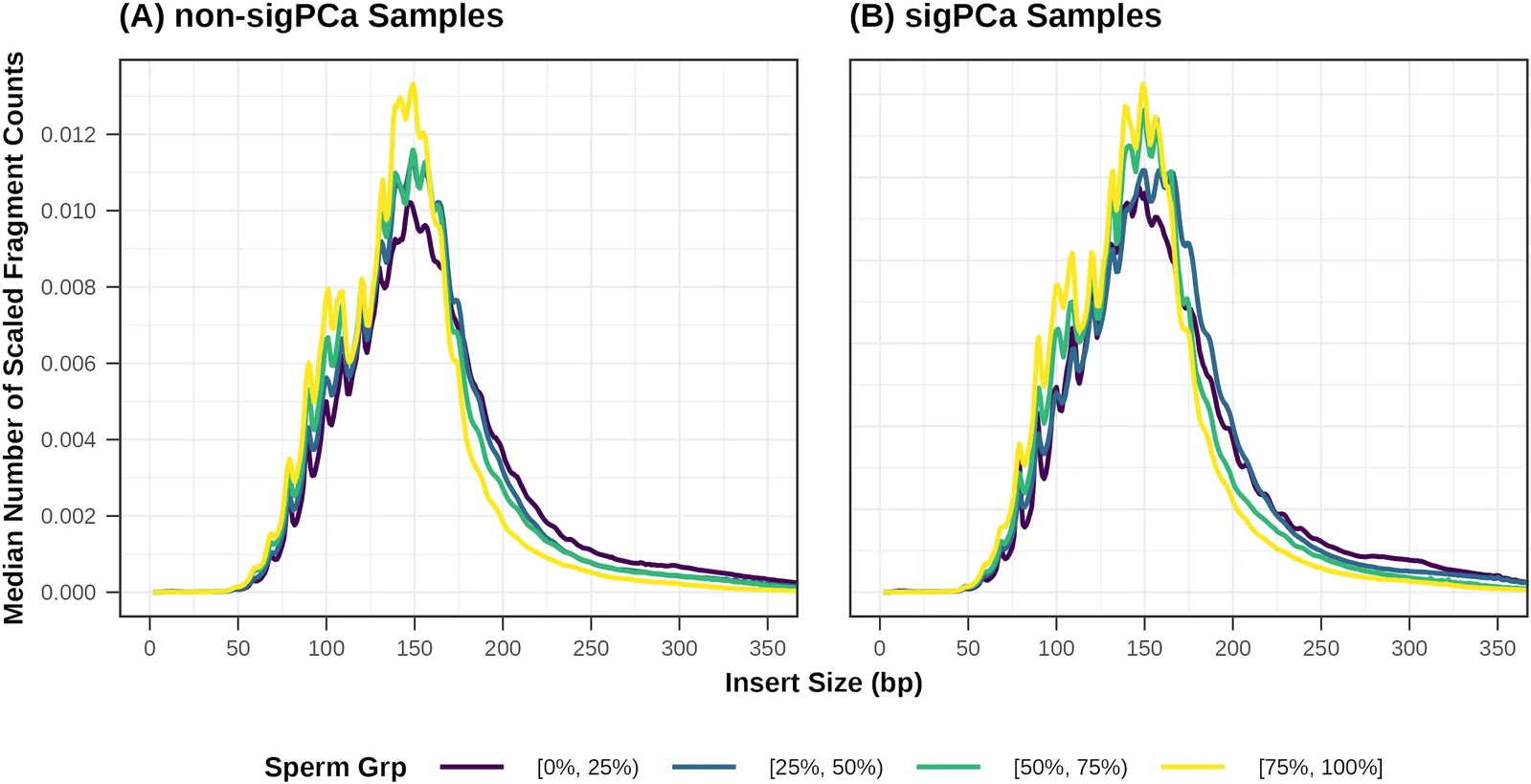
Insert size distribution shows that an increase in short fragments (< 125 bp) is associated with higher sperm fraction. A: Insert size for non-sigPCa. B: Insert size for sigPCa.

Interestingly, when insert size from sequencing reads was analyzed, it was observed that smaller fragments (< 125 bp) tended to increase with sperm amount and seemed to be associated with a peak around 100 bp. However, samples with no or little sperm signal (< 25%), including samples from men with and without a vasectomy, lacked this peak. These results may be due to the fact that during spermatogenesis there is a process of de-methylation [32,33] and such a state of hypomethylation is associated with shorter cfDNA fragments [34,35]. This phenomenon was observed in both non-sigPCa (see Fig 6A) and sigPCa samples (see Fig 6B).

### Methylome-wide unsupervised clustering

**Fig 7:**
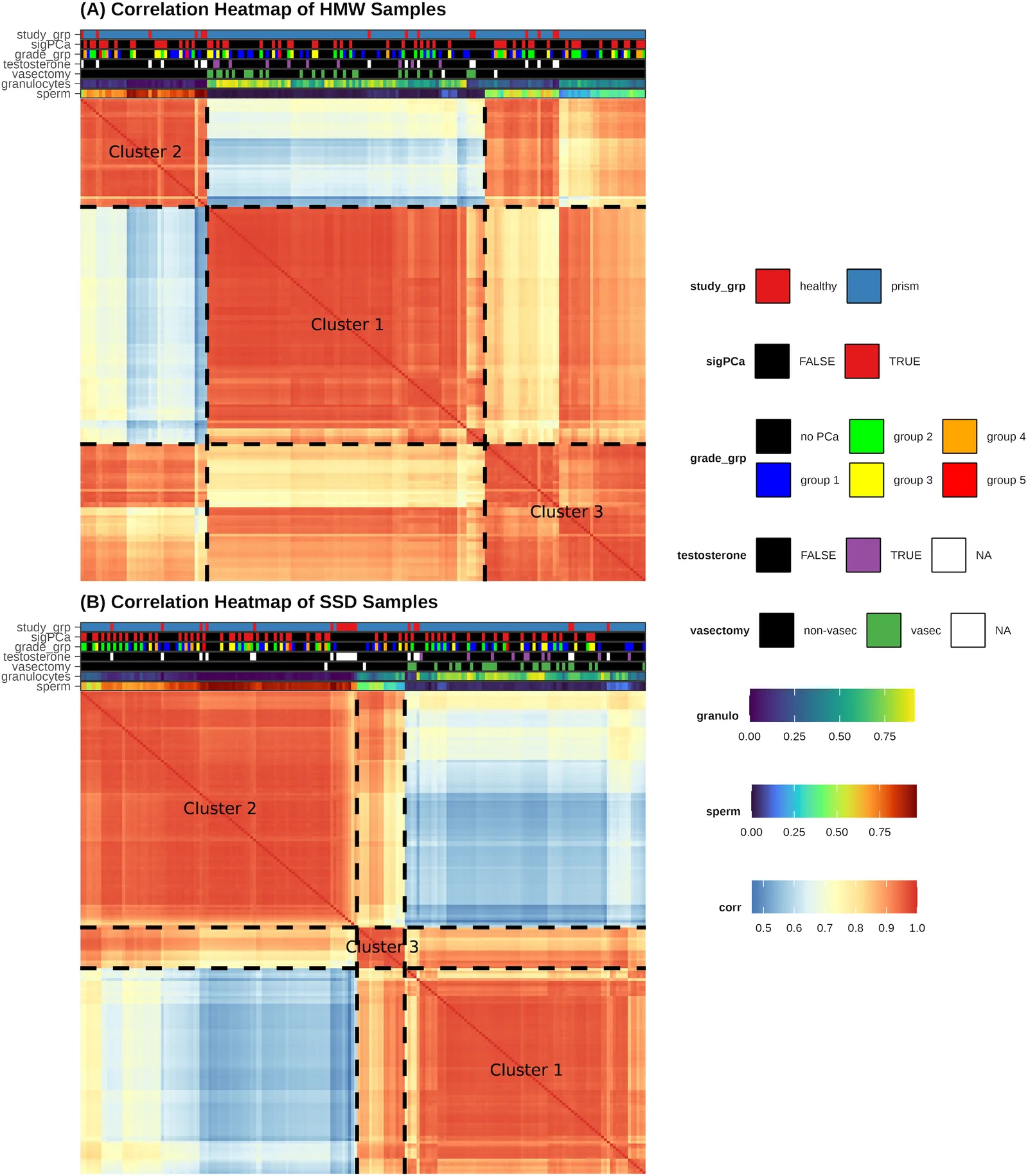
Unsupervised clustering based on beta values results in 3 clusters. A: HMW. B: SSD. In the horizontal bars at the top, missing data is colored white. In both HMW and SSD, samples cluster based on proportion of sperm and granulocyte. Note that in both cases the vasectomy and testosterone participants are in the no/low sperm cluster, but not all participants in the no/low sperm cluster were on testosterone or underwent vasectomy.

To understand the primary sources of variability methylome-wide, we calculated the pairwise correlation between all samples across all regions for beta-values and then performed unsupervised clustering for HMW and SSD separately (Fig 7). In both cases, the samples tended to separate into 3 clusters, two of which were highly uncorrelated (cluster label 1 and 2 in Fig 7A and 7B) and a third cluster (cluster label 3) that was modestly correlated with both of the other two. The clustering was not study driven (i.e., healthy individuals versus PRISM) as indicated by the top-most horizontal bar in Fig 7A and 7B. These clusters were also not associated with presence or absence of prostate cancer or GG (see the 2^nd^ and 3^rd^ horizontal bar in Fig 7A and 7B). They were, however, strongly associated with the estimated amount of sperm and granulocytes present (see last two horizontal bars in Fig 7A and 7B). Table 2 summarizes the cluster counts and average relative signals for sperm, granulocytes, and prostate for the three clusters. As expected, the men with vasectomy fell into the no/low sperm cluster, as did men on testosterone treatment (as indicated by the two horizontal bars labeled testosterone and vasectomy). The latter observation is consistent with previous literature that testosterone reduces sperm count [36,37]. However, the no/low sperm cluster was not exclusive to those types of participants and men that neither had a vasectomy nor were on testosterone treatment also strongly correlated. This clustering and correlations were not an artifact of the PRISM study as similar clustering were seen for the healthy individuals for clusters 1 and 2. None of the healthy individuals fell into cluster 3, likely due to the smaller sample size. Cluster 1, the “no/low sperm cluster”, forms the larger cluster in the HMW DNA which aligns with the fact that HMW has lower levels of sperm signal. Given these results, normalization for this background signal (sperm and granulocyte) would need to be performed if standard beta methods for methylation analysis were employed for the identification of biomarkers for sigPCa.

**Table 2:** Cluster details. Number of participants within each cluster are shown, in addition to average relative signals for sperm, granulocytes, and prostate tissues for healthy individuals and PRISM for both SSD and HMW.

		Cluster 1	Cluster 2	Cluster 3
**Healthy Individuals: SSD**	N	6	13	0
	Sperm	5.40%	86.10%	–
	Granulocyte	56.70%	2.80%	–
	Prostate	19.30%	4.00%	–
**Healthy Individuals: HMW**	N	5	6	4
	Sperm	2.40%	77.80%	44.80%
	Granulocyte	47.20%	2.80%	15.40%
	Prostate	19.30%	8.40%	16.40%
**PRISM: SSD**	N	75	80	16
	Sperm	4.20%	83.20%	38.60%
	Granulocyte	63.80%	7.80%	43.70%
	Prostate	8.30%	1.50%	2.50%
**PRISM: HMW**	N	85	35	48
	Sperm	2.20%	79.10%	40.20%
	Granulocyte	67.30%	8.50%	35.60%
	Prostate	5.50%	2.70%	4.80%

### Clinically significant prostate cancer biomarker discovery and classifier

While we first attempted to identify biomarkers that were relevant to sigPCa using traditional DMR approaches, it was readily apparent after our methylome-wide analysis that the dominant signals were from sperm and granulocytes and that any cancer signal was heavily confounded in the beta-values. In order to simplify our approach, we first focused on regions related to prostate cancer-related genes that were compiled based on an extensive literature search [38–53]. This consisted of 196 genes, and the methylome panel was subsetted to overlap these in ~4900 regions. We then created features from these regions as described in the Methods section which resulted in ~1.9 million features. Our empirical approach to preliminary feature filtering based on bootstrapping was then employed so that only features consistently demonstrating significant differentiation of non-sigPCa from sigPCa were retained as non-artifactual.

**Fig 8:**
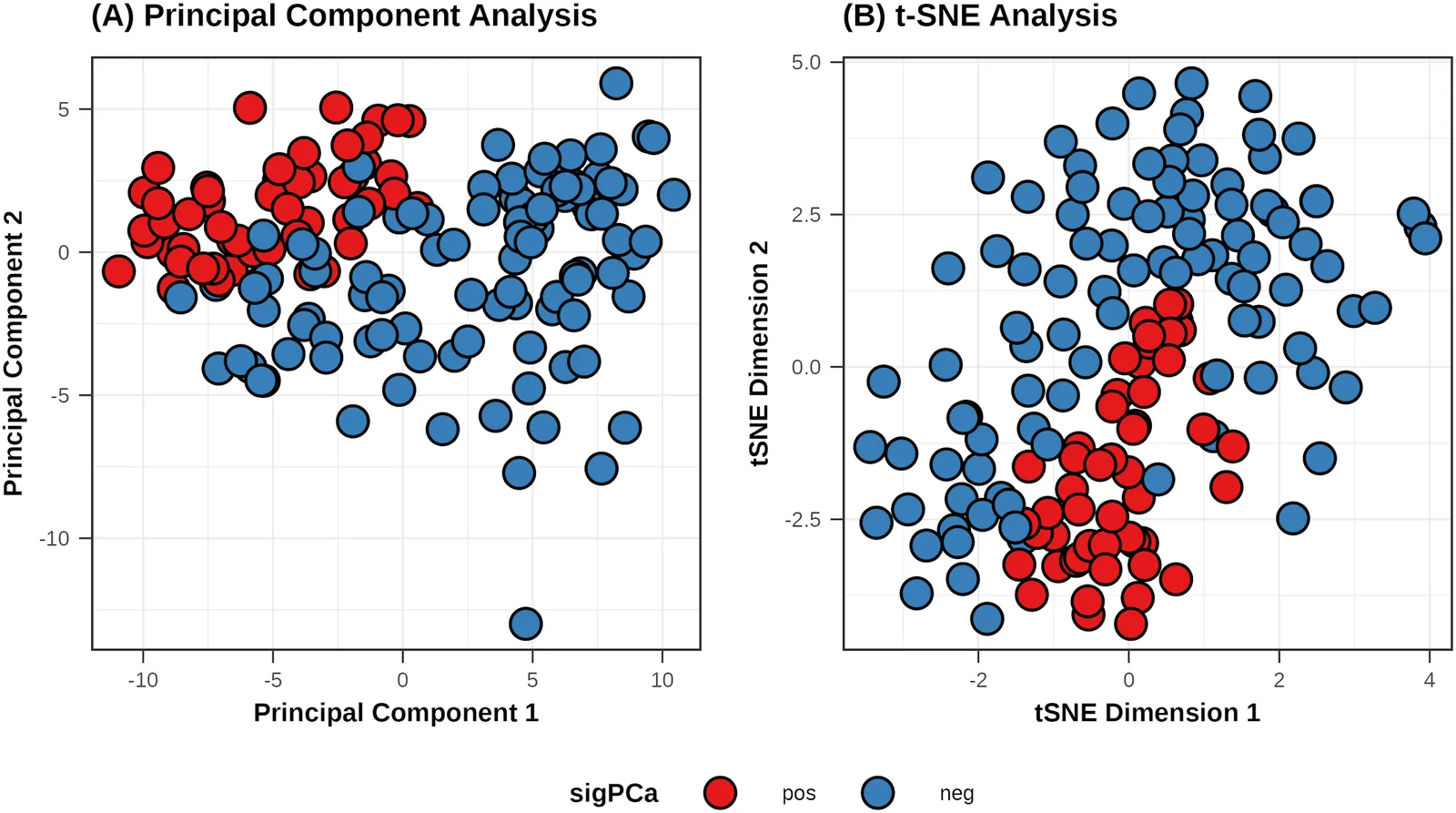
Dimension reduction using the selected features on the training data. A: Principal components 1 and 2. B: t-SNE.

**Fig 9:**
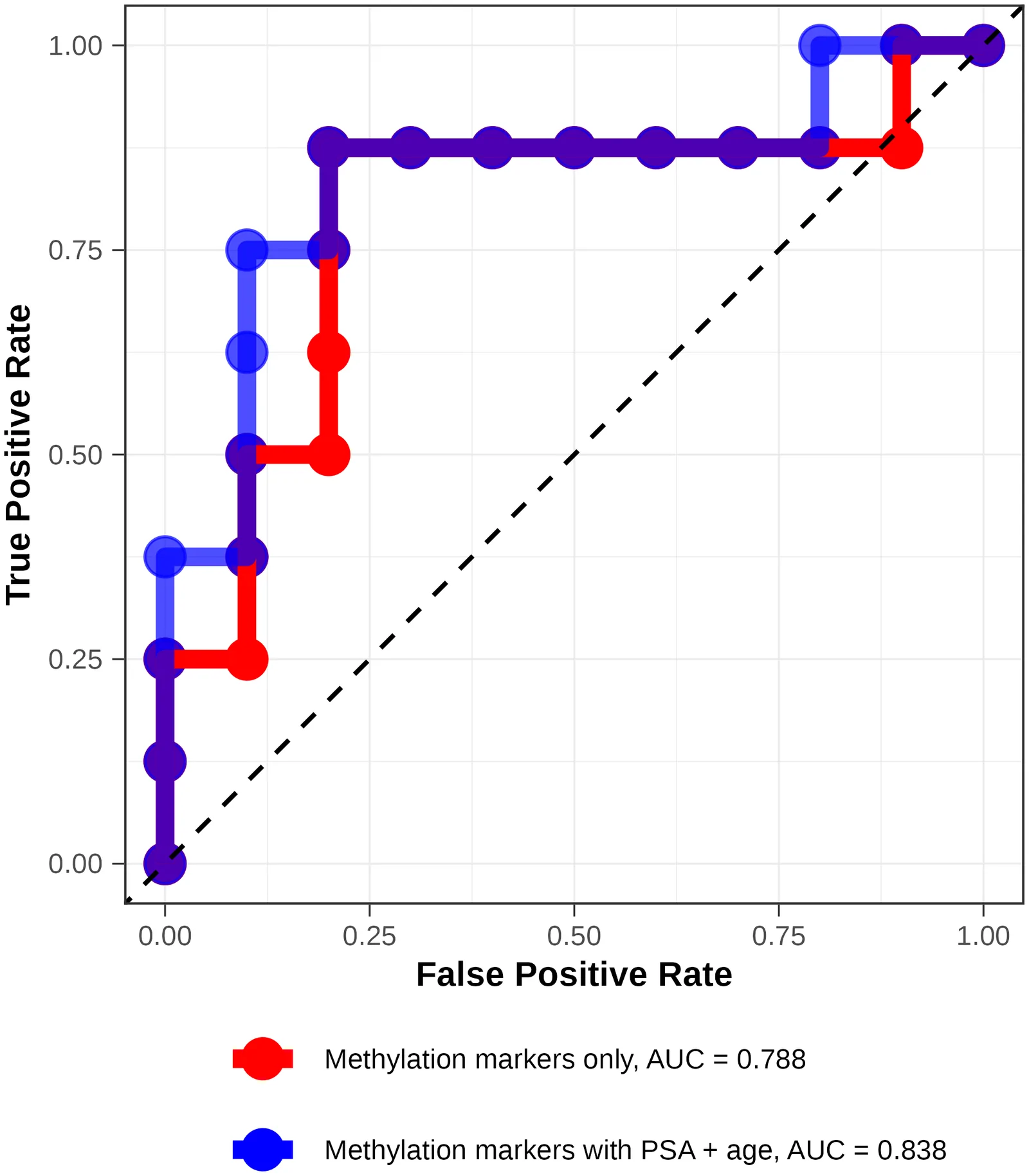
Area Under the Curve (AUC) from models without (red) and with age and PSA (blue).

Overall, this approach yielded 330 features from HMW cfDNA as potential biomarkers. When principal component analysis was performed on these features, we saw strong clustering of clinically significant vs clinically indolent/no cancer samples (Fig 8A). Additionally, t-SNE dimensionality reduction revealed a unique “horseshoe” effect where the non-sigPCa created a “U-shape” around the sigPCa (Fig 8B). A model was then trained as previously described and applied to a holdout set of 18 samples, yielding an AUC of 0.788. When age and PSA were added to the model, the AUC increased to 0.838 (see Fig 9).

## Discussion

Prostate-derived cfDNA is known to be at very low levels in blood, although one study demonstrated that post-biopsy, levels significantly increase [54]. While certainly less invasive than prostate biopsies, obtaining sufficient blood to detect prostate-derived cfDNA still requires medical intervention (i.e., phlebotomy). In this study we sought to investigate the diagnostic potential of seminal fluid since it can be self-collected in the comfort of one’s home. Additionally, since a previous clinical study demonstrated that 53.15% of men declined to participant when their semen specimen had to be delivered to the hospital within 2 hours of collection [55], one objective of this study was to determine if a mail-in option would improve participation rate. Results from this study suggest that having the flexible and discrete option of a mail-in test improves participation, since only 22% of enrolled patients in this study did not return a specimen.

In our previous study with healthy individuals, we demonstrated that there was an abundance of cfDNA in seminal fluid [10]. In the same study, we also demonstrated that the predominant fragment size of cfDNA in men having undergone vasectomy was > 1kb, with little to no nucleosomal DNA. In this larger study, we confirmed these findings with older men who were scheduled to undergo a prostate biopsy. Moreover, we also demonstrated that in men who had not undergone vasectomy, the size of the nucleosomal peak on Tapestation correlated to the amount of sperm derived cfDNA according to our methylation tissue deconvolution analysis. This was also reflected in the insert sizes where a notable increase in shorter fragments, manifesting as an additional “peak” in fragments around 100 bp, correlated with relative sperm content. Methylome-wide analysis confirmed that samples from men both with and without a vasectomy cluster together while correlating with estimated low sperm content and included men that were using testosterone which is known to decrease sperm (54, 55). In this cohort the normalized yield of cfDNA was more than 7 times than what was seen in the previous study, probably due to a large abundance of cfDNA from granulocytes in the majority of participants.

The reason for the higher granulocyte/lower sperm signal in the non-vasectomy cohort of this population compared to our previous cohort could be due to the fact that this is an older population, and thus the sperm fraction is likely to be lower. It could also be that since all participants in this cohort had been scheduled for a prostate biopsy (due to high PSA, abnormal DRE or other clinical symptoms potentially linked to prostate cancer) that there is a higher level of disease-related inflammation. Given the cfDNA yields are so much higher in this cohort, it points more to the latter hypothesis; that there is a higher amount of inflammation present in this cohort compared to our previous cohort.

Since approx. 85% of the DNA in mature sperm is bound to protamines and forms large toroidal structures [56,57], we purposely performed a high-speed spin to remove as much of the (sperm) background noise as possible. As was done in our previous study, we size- selected the cfDNA and analyzed the small size selected (SSD) cfDNA separately from the high molecular weight (HMW) cfDNA. As previously observed, we could boost the somatic cell signal by enriching for the HMW; however, in this population, there was vastly more granulocyte signal than prostate signal. It was surprising to see that in 39% (57/145) of non-vasectomy participants for which we had HMW methylation data, more than 50% of the cfDNA was derived from granulocytes. Since granulocyte signal was more highly enriched in the HMW than the SSD fraction, it is plausible that the source of this DNA is from NETosis, a unique form of programmed cell death in neutrophils that results in the formation and release of neutrophil extracellular traps (NETs). During NETosis, neutrophils expel decondensed chromatin packaged with antimicrobial proteins from their granules and cytoplasm into the extracellular space. While NETosis is part of the innate immune pathway, dysregulated or excessive NET formation can contribute to a variety of pathological conditions, including cancer [58,59]. Despite this very high granulocyte signal, the average prostate derived cfDNA was still high at 4.7% in HMW cfDNA. In 19 participants, more than 10% of HMW cfDNA was prostate derived.

Given the abundance of sperm cells in seminal fluid, one may have still expected to see sperm as the predominant source of nucleosomal (SSD) cfDNA in seminal fluid from men who had not undergone vasectomy. It was therefore surprising to see that for 45% (66/147) of the non-vasectomy participants for whom we had SSD methylation data, less than 50% of the cfDNA was derived from sperm. It was also surprising to see that in the 72 participants that had < 20% sperm in both SSD and HMW that 61.1% (44 participants) of them were neither on testosterone nor had undergone vasectomy. Fertility is known to decrease with age, but we did not see a correlation between age and sperm signal in the non-vasectomy group. There was also no correlation between normalized yield and granulocyte or sperm signal. The high variability of sperm signal could potentially be more indicative of the stage of sperm at death; for example, apoptosis of immature sperm would likely be associated with higher amounts of hypomethylated nucleosomal (SSD) cfDNA, while apoptosis of only mature sperm would be associated with lower amounts of hypomethylated nucleosomal (SSD) cfDNA and higher amounts of protamine associated cfDNA. Additional research into this is worthwhile, as it might offer insights into modes of infertility.

In DNA methylation analysis, the standard approach identifies differentially methylated loci (DML) at CpG, CHG, or CHH sites. Proximal DML are often aggregated into differentially methylated regions (DMRs), defined as genomic segments (ranging from single loci to multi-kilobase spans) exhibiting quantifiable methylation differences. Traditional DMR analysis relies on beta-values, which represent methylation proportions across multiple DNA fragments. This approach suffers from over-summarization, potentially confounding signals from heterogeneous cell populations when tissue-of-origin is either unknown or is not from the primary tissue of interest. Stackpole et al. [14] recently introduced alpha-values as an alternative metric, analyzing methylation fractions within individual DNA fragments using binary methylation calls. This method enhances sensitivity for detecting rare methylation events in complex mixtures (e.g., cell-free DNA). Despite these improvements, both beta- and alpha- values share a fundamental limitation: neither simultaneously captures locus-specific methylation proportions and fragment-level methylation patterns. Beta-values quantify site-specific methylation across fragments but ignore fragment-level coherence, while alpha-values quantify fragment-level methylation but reduce site-specific data to binary states. Critically, neither method accounts for the ordered spatial arrangement of methylated/unmethylated sites within a fragment – a key determinant of biological function. This over-summarization obscures potentially informative features that could be associated with specific tissues of origin.

In this study, by summarizing fragments based on methylation pattern similarity, we were able to overcome the predominant variability that is correlated with sperm and granulocytes. Both PCA (principal component analysis) and t-SNE revealed that these features can, not only cluster sigPCa from non-sigPCa samples, but also capture the sophisticated underlying structure. Clearly in PCA at least two principal components are required to achieve separation indicating that discriminative variability is distributed across multiple orthogonal dimensions (multiple components) and that these features likely need to work in combination with each other to capture interactions. The unique “horseshoe” pattern observed in the t-SNE plot supports that a complex and potentially non-linear infrastructure can achieve separation in the sample classes.

Despite this complicated structure, these features that have been derived to deconvolute their tissue of origin, could be used in an elastic-net logistic regression model to achieve fair performance on an independent test set. While an AUC of 0.788 is modest, the addition of age and PSA increased this to an AUC of 0.838. Given the observations from the dimensionality reduction analyses, we believe more sophisticated modeling and an increase in the training data could lead to more robust conclusions of superior model performance. However, by characterizing and understanding the methylome biological composition in seminal fluid through the addition of these clinical samples, we have demonstrated that biomarker features relevant to clinically significant prostate cancer could be identified.

This study corroborates our previous findings that somatic cell-derived cfDNA is highly abundant in seminal fluid. In this cohort of older men undergoing prostate biopsy for suspected prostate cancer, we observed markedly higher cfDNA yields and granulocyte-derived cfDNA signal compared to our earlier study of (younger) presumed healthy controls. Despite this elevated background, the mean fraction of prostate-derived cfDNA remained substantial, measuring 4.71% in the HMW fraction and 4.60% in the SSD fraction – levels that are notably higher than those typically observed in other liquid biopsy sources such as blood plasma. Standard methylation analysis using DMRs was unable to distinguish sigPCa from non-sigPCa, primarily due to high background noise and confounding effects arising from considerable inter-individual variation in sperm content. To overcome these challenges, we implemented a methylation analysis approach that addresses the over-summarization limitations of traditional methylation methodologies. In summary, this study provides the foundational principles for the development of an at-home seminal fluid collection test for the detection of sigPCa.

## Supporting information

S1 Fig Consort diagram for PRISM. (TIF)

S2 Fig Normalized yield as a function of sperm (top panel) and granulocyte fraction (bottom panel) in HMW DNA. (TIF)

S3 Fig Distribution of PSA (ng/mL) for N = 171 participants by GG. Participants with a vasectomy are the green “x’s”. Note that the plot does not show PSA > 18. There were three extreme PSAs, one in group 2, group 3, and group 4.
(TIF)

S1 Table Data dictionary. (CSV)

S2 Table PRISM participant clinical information. (CSV)

S3 Table HMW DNA tissue deconvolution data. Fraction of each tissue type by participant ID.
(CSV)

S4 Table SSD DNA tissue deconvolution data. Fraction of each tissue type by participant ID.
(CSV)

S5 Table SSD insert sizes. Number of reads for each insert size (2 bp-400 bp), by participant ID.
(CSV)

S6 Table HMW methylome correlations. Correlation coefficients of beta values, for each PRISM participant against every other PRISM participant, and itself, for HMW cfDNA.
(CSV)

S7 Table SSD methylome correlations. Correlation coefficients of beta values, for each PRISM participant against every other PRISM participant, and itself, for SSD cfDNA.
(CSV)

S8 Table Principal component analysis results for training data. Differentially methylated pattern (DMP) principal component 1 (pc1) and principal component 2 (pc2) values for PRISM participants in the training dataset.
(CSV)

S9 Table TNSE analysis results for training data. Differentially methylated pattern (DMP) dimensionality 1 (dim1) and dimensionality 2 (dim2) values for PRISM participants in the training dataset.
(CSV)

S10 Table Receiver operating curve (ROC) data. False positive rate (fpr) and true positive rate (tpr) for the hold out test set, demonstrating the best performance is a fpr of 0.2 and a tpr of 0.875.
(CSV)

S11 Table Receiver operating curve (ROC) data with age and PSA. False positive rate (fpr) and true positive rate (tpr) for the hold out test set, demonstrating the best performance is a fpr of 0.2 and a tpr of 0.875.
(CSV)

S12 Table PRISM cfDNA yield. Column A, participant ID; Column B, total yield from extraction; Column C, volume of seminal plasma into extraction; Column D, normalized yield per ml plasma.
(CSV)
