# Supplementary material for: Uncovering the diagnostic potential of seminal fluid beyond fertility: cfDNA methylation analysis for the detection of clinically significant prostate cancer
Source: PLoS One. 2026 Sep 9;21(9):e0356387. doi: 10.1371/journal.pone.0356387 (PMC13557413; doi:10.1371/journal.pone.0356387)
Supplement: Supplementary file 1 [file pone.0356387.s017.docx]

Dear Reviewers,

Thank you for your questions and comments on our manuscript entitled "Uncovering the diagnostic potential of seminal fluid beyond fertility: cfDNA methylation analysis for the detection of clinically significant prostate cancer". Please see below for our responses:

Reviewer #1: Authors should recognize that the idea is not new. In 2018 Lazzeri and co-workers by the first presented the possibility of using seminal fluid for diagnosis of PCa. In their paper they investigated the role of tumor cell in the seminal fluid showing the potential clinical application. Authors should recognize the reference in their paper (Lazzeri, M.; Colombo, F.; Chiereghin, C.; Buffi, N.; Casale, P.; Hurle, R.; Saita, A.; Lughezzani, G.; Asselta, R.; Colombo, P.; et al. Pd06-10 Liquid Biopsy by Prostate-Derived Tumor Cells Enriched From Seminal Fluid (Sf): The Semen Prostate Cancer Tumor Elements (Spectre) Project. J. Urol. 2018, 199, e154–e155).

Response: This reference has been added to the introduction.

Reviewer #1: The idea of cfDNA in seminal fluid is really interesting but a correlation with cfDNA from blood could be used complementary. Did authors investigated the topic. Please address the issue using the following references: Corbetta M, Chiereghin C, De Simone I, Soldà G, Zuradelli M, Giunta M, Lughezzani G, Buffi NM, Hurle R, Saita A, Casale P, Asselta R, Lazzeri M, Guazzoni G, Duga S. Post-Biopsy Cell-Free DNA From Blood: An Open Window on Primary Prostate Cancer Genetics and Biology. Front Oncol. 2021 May 24;11:654140. doi: 10.3389/fonc.2021.654140. PMID: 34109115; PMCID: PMC8181420.

Response: We now reference this study in the discussion. We did not investigate the use of cfDNA in blood because we were interested in developing a test whereby the specimen could be self-collected at home. The benefits of such are obvious and combining with cfDNA would eliminate the ability to self-collect a specimen at home.

Reviewer #1: Finally, authors reported a “Of 276 enrolled participants, 60 (22%) did not provide a specimen.” Saitta and co-workers previously addressed the issue reaching an opposite result. Indeed before addressing whether the liquid biopsy of SF is a valid and clinically useful option, it is important to evaluate the patients’ willingness to donate and provide a SF sample. Saitta and co-workers reported about 760 patients with localized PCa, who were scheduled for whole gland treatment (RP or radiotherapy) and were proposed to provide their SF. Of 760 patients, 404 (53.15%) declined to participate. Among the 356 patients who accepted to participate, 139 (39%) were able to collect a SF sample. Considering 217 patients who did not collected the SF, 130 (59.9%) reported that failure was due to psychogenic inhibition at the time of masturbation, 33 (15.2%) suffered from erectile dysfunction, and 54 (24.9%) had retrograde ejaculation due to alpha blocker treatment (Saitta C, De Simone I, Fasulo V, Corbetta M, Duga S, Chiereghin C, Colombo FS, Benetti A, Contieri R, Avolio PP, Uleri A, Saita A, Guazzoni GF, Hurle R, Colombo P, Buffi NM, Casale P, Lughezzani G, Asselta R, Soldà G, Lazzeri M. Evaluation of Semen Self-Sampling Yield Predictors and CTC Isolation by Multi-Color Flow Cytometry for Liquid Biopsy of Localized Prostate Cancer. Cancers (Basel). 2023 May 9;15(10):2666. doi: 10.3390/cancers15102666. PMID: 37345004; PMCID: PMC10216665.).

Response: This reference has been added to the discussion section in addition to some text highlighting why the participation rate in our study was so much higher than the study be Saitta et al.

Best,

-Kim Clark-Langone
