# Supplementary material for: Uncovering the diagnostic potential of seminal fluid beyond fertility: cfDNA methylation analysis for the detection of clinically significant prostate cancer
Source: PLoS One. 2026 Sep 9;21(9):e0356387. doi: 10.1371/journal.pone.0356387 (PMC13557413; doi:10.1371/journal.pone.0356387)
Supplement: Peer review history

# Peer review: Decision Letter 0

**DOI:** 10.1371/journal.pone.0356387.r001

**Authors:** Elingarami Sauli

23 Apr 2026

Dear Dr. Kim,

Thank you for submitting your manuscript to PLOS ONE. After careful consideration, we feel that it has merit but does not fully meet PLOS ONE’s publication criteria as it currently stands. Therefore, we invite you to submit a revised version of the manuscript that addresses the points raised during the review process.

I forward our regrets to the authors for the very delayed feedback regarding this submission. Kindly respond to highlighted reviewer comments. Also highlight reproducibility of this diagnostic potential for prostate cancer. Please also clearly indicate all limitations in your study, starting with the methodological approach to your obtained results.

Please submit your revised manuscript by Jun 07 2026 11:59PM. If you will need more time than this to complete your revisions, please reply to this message or contact the journal office at plosone@plos.org. When you're ready to submit your revision, log on to https://www.editorialmanager.com/pone/ and select the 'Submissions Needing Revision' folder to locate your manuscript file.

- A letter that responds to each point raised by the academic editor and reviewer(s). You should upload this letter as a separate file labeled 'Response to Reviewers'.
- A marked-up copy of your manuscript that highlights changes made to the original version. You should upload this as a separate file labeled 'Revised Manuscript with Track Changes'.
- An unmarked version of your revised paper without tracked changes. You should upload this as a separate file labeled 'Manuscript'.

If you would like to make changes to your financial disclosure, please include your updated statement in your cover letter. Guidelines for resubmitting your figure files are available below the reviewer comments at the end of this letter.

If applicable, we recommend that you deposit your laboratory protocols in protocols.io to enhance the reproducibility of your results. Protocols.io assigns your protocol its own identifier (DOI) so that it can be cited independently in the future. For instructions see: https://journals.plos.org/plosone/s/submission-guidelines#loc-laboratory-protocols. Additionally, PLOS ONE offers an option for publishing peer-reviewed Lab Protocol articles, which describe protocols hosted on protocols.io. Read more information on sharing protocols at https://plos.org/protocols?utm_medium=editorial-email&utm_source=authorletters&utm_campaign=protocols.

As the corresponding author, your ORCID iD is verified in the submission system and will appear in the published article. PLOS supports the use of ORCID, and we encourage all coauthors to register for an ORCID iD and use it as well. Please encourage your coauthors to verify their ORCID iD within the submission system before final acceptance, as unverified ORCID iDs will not appear in the published article. *Only*  the individual author can complete the verification step; PLOS staff *cannot*  verify ORCID iDs on behalf of authors.

We look forward to receiving your revised manuscript.

Kind regards,

Elingarami Sauli, PhD

Academic Editor

PLOS One

Journal requirements:

When submitting your revision, we need you to address these additional requirements.

1. Please ensure that your manuscript meets PLOS ONE's style requirements, including those for file naming. The PLOS ONE style templates can be found at

https://journals.plos.org/plosone/s/file?id=wjVg/PLOSOne_formatting_sample_main_body.pdf and

https://journals.plos.org/plosone/s/file?id=ba62/PLOSOne_formatting_sample_title_authors_affiliations.pdf

2. Thank you for stating the following in the Competing Interests section:

“I have read the journal's policy and the authors of the manuscript have the following competing interests: J.F.S., S.B., H.A. and K.M.C.L. are current employees of Fellow Health and have stock ownership. J.G., T.M. and D.C. are consultants for Fellow Health and have stock ownership. S.H., J.C.H. and K.M.C.L. are inventors a non-provisional patent application related to cfDNA isolation from seminal plasma. J.G., T.M., D.C. and K.M.C.L are inventors a provisional patent application related to methylation pattern analysis.”

Please confirm that this does not alter your adherence to all PLOS ONE policies on sharing data and materials, by including the following statement: "This does not alter our adherence to  PLOS ONE policies on sharing data and materials.” (as detailed online in our guide for authors http://journals.plos.org/plosone/s/competing-interests).  If there are restrictions on sharing of data and/or materials, please state these. Please note that we cannot proceed with consideration of your article until this information has been declared.

Please include your updated Competing Interests statement in your cover letter; we will change the online submission form on your behalf.

3. We note that you have indicated that there are restrictions to data sharing for this study. For studies involving human research participant data or other sensitive data, we encourage authors to share de-identified or anonymized data. However, when data cannot be publicly shared for ethical reasons, we allow authors to make their data sets available upon request. For information on unacceptable data access restrictions, please see http://journals.plos.org/plosone/s/data-availability#loc-unacceptable-data-access-restrictions.

Before we proceed with your manuscript, please address the following prompts:

a) If there are ethical or legal restrictions on sharing a de-identified data set, please explain them in detail (e.g., data contain potentially identifying or sensitive patient information, data are owned by a third-party organization, etc.) and who has imposed them (e.g., a Research Ethics Committee or Institutional Review Board, etc.). Please also provide contact information for a data access committee, ethics committee, or other institutional body to which data requests may be sent.

b) If there are no restrictions, please upload the minimal anonymized data set necessary to replicate your study findings to a stable, public repository and provide us with the relevant URLs, DOIs, or accession numbers. Please see http://www.bmj.com/content/340/bmj.c181.long for guidelines on how to de-identify and prepare clinical data for publication. For a list of recommended repositories, please see https://journals.plos.org/plosone/s/recommended-repositories. You also have the option of uploading the data as Supporting Information files, but we would recommend depositing data directly to a data repository if possible.

Please update your Data Availability statement in the submission form accordingly.

4. We note that there is identifying data in the Supporting tables 2, 3, 4, 5, 6, 7, 8, 9 and 12 . Due to the inclusion of these potentially identifying data, we have removed this file from your file inventory. Prior to sharing human research participant data, authors should consult with an ethics committee to ensure data are shared in accordance with participant consent and all applicable local laws.

Data sharing should never compromise participant privacy. It is therefore not appropriate to publicly share personally identifiable data on human research participants. The following are examples of data that should not be shared:

-Name, initials, physical address

-Ages more specific than whole numbers

-Internet protocol (IP) address

-Specific dates (birth dates, death dates, examination dates, etc.)

-Contact information such as phone number or email address

-Location data

-ID numbers that seem specific (long numbers, include initials, titled “Hospital ID”) rather than random (small numbers in numerical order)

Data that are not directly identifying may also be inappropriate to share, as in combination they can become identifying. For example, data collected from a small group of participants, vulnerable populations, or private groups should not be shared if they involve indirect identifiers (such as sex, ethnicity, location, etc.) that may risk the identification of study participants.

Additional guidance on preparing raw data for publication can be found in our Data Policy (https://journals.plos.org/plosone/s/data-availability#loc-human-research-participant-data-and-other-sensitive-data) and in the following article: http://www.bmj.com/content/340/bmj.c181.long.

Please remove or anonymize all personal information (<specific identifying information in file to be removed>), ensure that the data shared are in accordance with participant consent, and re-upload a fully anonymized data set. Please note that spreadsheet columns with personal information must be removed and not hidden as all hidden columns will appear in the published file.

If the reviewer comments include a recommendation to cite specific previously published works, please review and evaluate these publications to determine whether they are relevant and should be cited. There is no requirement to cite these works unless the editor has indicated otherwise.

Please review your reference list to ensure that it is complete and correct. If you have cited papers that have been retracted, please include the rationale for doing so in the manuscript text, or remove these references and replace them with relevant current references. Any changes to the reference list should be mentioned in the rebuttal letter that accompanies your revised manuscript. If you need to cite a retracted article, indicate the article’s retracted status in the References list and also include a citation and full reference for the retraction notice.

[Note: HTML markup is below. Please do not edit.]

Reviewers' comments:

Reviewer's Responses to Questions

**Comments to the Author**

1. Is the manuscript technically sound, and do the data support the conclusions?

Reviewer #1: Yes

**********

2. Has the statistical analysis been performed appropriately and rigorously?

Reviewer #1: Yes

**********

3. Have the authors made all data underlying the findings in their manuscript fully available?

The PLOS Data policy

Reviewer #1: Yes

**********

4. Is the manuscript presented in an intelligible fashion and written in standard English?

Reviewer #1: Yes

**********

Reviewer #1: I read with great interest the paper about the potential role of cfDNA in seminal fluid for diagnosis of Prostate Cancer (PCA). Although the sample size remains small and the study design could meet several drawbacks in clinical application, it deserves attention.

I have some concerns and suggestions.

Authors should recognize that the idea is not new. In 2018 Lazzeri and co-workers by the first presented the possibility of using seminal fluid for diagnosis of PCa. In their paper they investigated the role of tumor cell in the seminal fluid showing the potential clinical application. Authors should recognise the reference in their paper (Lazzeri, M.; Colombo, F.; Chiereghin, C.; Buffi, N.; Casale, P.; Hurle, R.; Saita, A.; Lughezzani, G.; Asselta, R.; Colombo, P.; et al. Pd06-10 Liquid Biopsy by Prostate-Derived Tumor Cells Enriched From Seminal Fluid (Sf): The Semen Prostate Cancer Tumor Elements (Spectre) Project. J. Urol. 2018, 199, e154–e155)

The idea of cfDNA in seminal fluid is really interesting but a correlation with cfDNA from blood could be used complementary. Did authors investigated the topic. Please address the issue using the following references: Corbetta M, Chiereghin C, De Simone I, Soldà G, Zuradelli M, Giunta M, Lughezzani G, Buffi NM, Hurle R, Saita A, Casale P, Asselta R, Lazzeri M, Guazzoni G, Duga S. Post-Biopsy Cell-Free DNA From Blood: An Open Window on Primary Prostate Cancer Genetics and Biology. Front Oncol. 2021 May 24;11:654140. doi: 10.3389/fonc.2021.654140. PMID: 34109115; PMCID: PMC8181420.

Finally, authors reported a “Of 276 enrolled participants, 60 (22%) did not provide a specimen.” Saitta and co-workers previously addressed the issue reaching an opposite result. Indeed before addressing whether the liquid biopsy of SF is a valid and clinically useful option, it is important to evaluate the patients’ willingness to donate and provide a SF sample. Saitta and co-workers reported about 760 patients with localized PCa, who were scheduled for whole gland treatment (RP or radiotherapy) and were proposed to provide their SF. Of 760 patients, 404 (53.15%) declined to participate. Among the 356 patients who accepted to participate, 139 (39%) were able to collect a SF sample. Considering 217 patients who did not collected the SF, 130 (59.9%) reported that failure was due to psychogenic inhibition at the time of masturbation, 33 (15.2%) suffered from erectile dysfunction, and 54 (24.9%) had retrograde ejaculation due to alpha blocker treatment (Saitta C, De Simone I, Fasulo V, Corbetta M, Duga S, Chiereghin C, Colombo FS, Benetti A, Contieri R, Avolio PP, Uleri A, Saita A, Guazzoni GF, Hurle R, Colombo P, Buffi NM, Casale P, Lughezzani G, Asselta R, Soldà G, Lazzeri M. Evaluation of Semen Self-Sampling Yield Predictors and CTC Isolation by Multi-Color Flow Cytometry for Liquid Biopsy of Localized Prostate Cancer. Cancers (Basel). 2023 May 9;15(10):2666. doi: 10.3390/cancers15102666. PMID: 37345004; PMCID: PMC10216665.).

**********

what does this mean?). If published, this will include your full peer review and any attached files.

If you choose “no”, your identity will remain anonymous but your review may still be made public.

**Do you want your identity to be public for this peer review?**  For information about this choice, including consent withdrawal, please see our Privacy Policy

Reviewer #1: No

**********

[NOTE: If reviewer comments were submitted as an attachment file, they will be attached to this email and accessible via the submission site. Please log into your account, locate the manuscript record, and check for the action link "View Attachments". If this link does not appear, there are no attachment files.]

To ensure your figures meet our technical requirements, please review our figure guidelines: https://journals.plos.org/plosone/s/figures

You may also use PLOS’s free figure tool, NAAS, to help you prepare publication quality figures: https://journals.plos.org/plosone/s/figures#loc-tools-for-figure-preparation.

NAAS will assess whether your figures meet our technical requirements by comparing each figure against our figure specifications.

# Author comment: Author response to Decision Letter 1

**DOI:** 10.1371/journal.pone.0356387.r002

30 Jul 2026

Reviewer #1: Authors should recognize that the idea is not new. In 2018 Lazzeri and co-workers by the first presented the possibility of using seminal fluid for diagnosis of PCa. In their paper they investigated the role of tumor cell in the seminal fluid showing the potential clinical application. Authors should recognize the reference in their paper (Lazzeri, M.; Colombo, F.; Chiereghin, C.; Buffi, N.; Casale, P.; Hurle, R.; Saita, A.; Lughezzani, G.; Asselta, R.; Colombo, P.; et al. Pd06-10 Liquid Biopsy by Prostate-Derived Tumor Cells Enriched From Seminal Fluid (Sf): The Semen Prostate Cancer Tumor Elements (Spectre) Project. J. Urol. 2018, 199, e154–e155).

Response: This reference has been added to the introduction.

Reviewer #1: The idea of cfDNA in seminal fluid is really interesting but a correlation with cfDNA from blood could be used complementary. Did authors investigated the topic. Please address the issue using the following references: Corbetta M, Chiereghin C, De Simone I, Soldà G, Zuradelli M, Giunta M, Lughezzani G, Buffi NM, Hurle R, Saita A, Casale P, Asselta R, Lazzeri M, Guazzoni G, Duga S. Post-Biopsy Cell-Free DNA From Blood: An Open Window on Primary Prostate Cancer Genetics and Biology. Front Oncol. 2021 May 24;11:654140. doi: 10.3389/fonc.2021.654140. PMID: 34109115; PMCID: PMC8181420.

Response: We now reference this study in the discussion. We did not investigate the use of cfDNA in blood because we were interested in developing a test whereby the specimen could be self-collected at home. The benefits of such are obvious and combining with cfDNA would eliminate the ability to self-collect a specimen at home.

Reviewer #1: Finally, authors reported a “Of 276 enrolled participants, 60 (22%) did not provide a specimen.” Saitta and co-workers previously addressed the issue reaching an opposite result. Indeed before addressing whether the liquid biopsy of SF is a valid and clinically useful option, it is important to evaluate the patients’ willingness to donate and provide a SF sample. Saitta and co-workers reported about 760 patients with localized PCa, who were scheduled for whole gland treatment (RP or radiotherapy) and were proposed to provide their SF. Of 760 patients, 404 (53.15%) declined to participate. Among the 356 patients who accepted to participate, 139 (39%) were able to collect a SF sample. Considering 217 patients who did not collected the SF, 130 (59.9%) reported that failure was due to psychogenic inhibition at the time of masturbation, 33 (15.2%) suffered from erectile dysfunction, and 54 (24.9%) had retrograde ejaculation due to alpha blocker treatment (Saitta C, De Simone I, Fasulo V, Corbetta M, Duga S, Chiereghin C, Colombo FS, Benetti A, Contieri R, Avolio PP, Uleri A, Saita A, Guazzoni GF, Hurle R, Colombo P, Buffi NM, Casale P, Lughezzani G, Asselta R, Soldà G, Lazzeri M. Evaluation of Semen Self-Sampling Yield Predictors and CTC Isolation by Multi-Color Flow Cytometry for Liquid Biopsy of Localized Prostate Cancer. Cancers (Basel). 2023 May 9;15(10):2666. doi: 10.3390/cancers15102666. PMID: 37345004; PMCID: PMC10216665.).

Response: This reference has been added to the discussion section in addition to some text highlighting why the participation rate in our study was so much higher than the study be Saitta et al.

Attachment Submitted filename: Response to Reviewers.docx

# Editor report: Decision Letter 1

**DOI:** 10.1371/journal.pone.0356387.r003

**Authors:** Elingarami Sauli

3 Aug 2026

Uncovering the diagnostic potential of seminal fluid beyond fertility: cfDNA methylation analysis for the detection of clinically significant prostate cancer.

PONE-D-25-46561R1

Dear Dr. Kim,

We’re pleased to inform you that your manuscript has been judged scientifically suitable for publication and will be formally accepted for publication once it meets all outstanding technical requirements.

Within one week, you’ll receive an e-mail detailing the required amendments. When these have been addressed, you’ll receive a formal acceptance letter and your manuscript will be scheduled for publication.

An invoice will be generated when your article is formally accepted. Please note, if your institution has a publishing partnership with PLOS and your article meets the relevant criteria, all or part of your publication costs will be covered. Please make sure your user information is up-to-date by logging into Editorial Manager at Editorial Manager® and clicking the ‘Update My Information' link at the top of the page. For questions related to billing, please contact billing support.

If your institution or institutions have a press office, please notify them about your upcoming paper to help maximize its impact. If they’ll be preparing press materials, please inform our press team as soon as possible -- no later than 48 hours after receiving the formal acceptance. Your manuscript will remain under strict press embargo until 2 pm Eastern Time on the date of publication. For more information, please contact onepress@plos.org.

Kind regards,

Elingarami Sauli, PhD

Academic Editor

PLOS One

Additional Editor Comments (optional):

I have reviewed the authors' responses to reviewer 1 and i'm satisfied with received responses regarding the raised minor comments. To my satisfaction, this submission can now be accepted for publication.

Reviewers' comments:

# Editor report: Acceptance letter

**DOI:** 10.1371/journal.pone.0356387.r004

**Authors:** Elingarami Sauli

PONE-D-25-46561R1

PLOS One

Dear Dr. Clark-Langone,

I'm pleased to inform you that your manuscript has been deemed suitable for publication in PLOS One. Congratulations! Your manuscript is now being handed over to our production team.

At this stage, our production department will prepare your paper for publication. This includes ensuring the following:

* All references, tables, and figures are properly cited

* All relevant supporting information is included in the manuscript submission,

* There are no issues that prevent the paper from being properly typeset

You will receive further instructions from the production team, including instructions on how to review your proof when it is ready. Please keep in mind that we are working through a large volume of accepted articles, so please give us a few days to review your paper and let you know the next and final steps.

Lastly, if your institution or institutions have a press office, please let them know about your upcoming paper now to help maximize its impact. If they'll be preparing press materials, please inform our press team within the next 48 hours. Your manuscript will remain under strict press embargo until 2 pm Eastern Time on the date of publication. For more information, please contact onepress@plos.org.

You will receive an invoice from PLOS for your publication fee after your manuscript has reached the completed accept phase. If you receive an email requesting payment before acceptance or for any other service, this may be a phishing scheme. Learn how to identify phishing emails and protect your accounts at https://explore.plos.org/phishing.

If we can help with anything else, please email us at customercare@plos.org.

Thank you for submitting your work to PLOS One and supporting open access.

Kind regards,

PLOS One Editorial Office Staff

on behalf of

Dr. Elingarami Sauli

Academic Editor

PLOS One
